# Comparison of radiogrammetrical metacarpal indices in children and reference data from the First Zurich Longitudinal Study

**DOI:** 10.1007/s00247-012-2390-6

**Published:** 2012-06-06

**Authors:** David D. Martin, Conrad Heckmann, Julia Neuhof, Oskar G. Jenni, Michael B. Ranke, Gerhard Binder

**Affiliations:** 1 Pediatric Endocrinology and Diabetology, University Children’s Hospital Tübingen, Hoppe-Seyler-Str. 1, 72076 Tuebingen, Germany; 2 Child Development Centre, University Children’s Hospital, Steinwiesstrasse 75, 8032 Zurich, Switzerland

**Keywords:** Radiogrammetry, Densitometry, Metacarpal dimensions, Skeletal growth, Child, Reference values

## Abstract

**Background:**

A number of radiogrammetrical metacarpal indices are in use, some of which have been adapted for children.

**Objective:**

The purpose of this study was to compare four known indices—bone mineral density (BMD), relative cortical area, Exton-Smith index, bending breaking resistance index—and the more recently defined pediatric bone index (PBI) according to the two criteria of minimum height dependence and minimum variability in children of equal bone age.

**Materials and methods:**

A total of 3,121 left-hand radiographs from 231 healthy Caucasian children ranging in age from 3 to 19 years old were analysed using BoneXpert®, a programme for automatic analysis of hand radiographs and assessment of bone age.

**Results:**

Dependence on height for chronological age or bone age and the mean relative standard deviation were lowest in the PBI for both genders pooled. The differences in height dependence were statistically significant and are shown to be clinically relevant. Reference data for PBI are presented.

**Conclusion:**

PBI may be a better indicator than BMD for bone health in children; however, verification in a clinical group is needed.

## Introduction

Bone densitometry is increasingly used for following the course of various paediatric disorders and treatments [1–9]. While still largely the domain of dual-energy X-ray densitometry (DXA) and peripheral quantitative CT (pQCT), with the advance of computerised image analysis over the past decade the field has again become accessible for radiogrammetrical techniques. These have the advantages of being inexpensive and convenient to use in children as well as involving lower radiation exposure [4, 10] and involve lower radiation exposure than both DXA and pQCT [11]. Methods that perform bone age assessment and radiogrammetrical measurements in a single run, like the one presented in this paper, pose a further advantage of the radiogrammetrical option. Since bone densitometry measurements are more dependent on skeletal maturity than on chronological age, a bone age-estimate should accompany any densitometry measurement in children suspected of having a developmental disorder [12]. Moreover, radiographs are readily available for retrospective radiogrammetrical studies. The precision of radiogrammetry is as good as or better than that of absorptiometrical techniques [7, 13–16]. It is based on the simplifying assumption that the density of cortical bone is constant and that cortical mass and volume are therefore interchangeable measurements. This assumption is valid in all but a few rare clinical conditions. The relative merits of radiogrammetry, DXA and pQCT are further discussed by Thodberg at al. [11]. Radio grammetrical measures are generally based on metacarpal width (W), length (L) and cortical thickness (T) and the derived measurement of medullary diameter, M = W−2·T.

The primary concern of bone densitometry is bone strength. Bone mass has been shown to account for 75–85% of the variance in the ultimate strength [17, 18]. An obvious point of departure in designing a radiogrammetrical index for long bones therefore is the cross-sectional area A = π(W/2)^2^ – π((W/2) – T)^2^ = π T·W(1 – T/W). To date the most widely used radiogrammetrical index has been area bone mineral density (BMD), which is defined as the cortical volume of a metacarpal bone divided by the area onto which it projects in the frontal view: π·T·(1–T/W) = A/W and is therefore measured in units of length [mm] [4, 5, 8, 9, 13, 15, 19–24]. BMD is often also referred to as DXR-BMD to distinguish it from area bone mineral density measured by DXA (DXA-BMD), which is measured in unit g/cm^2^. These two measurements in turn must be distinguished from volumetric bone mineral density as measured by pQCT, which is given in units of true density, i.e. g/cm^3^. Another radiogrammetrical index is the dimensionless relative cortical area (RCA, defined here as (W^2^−M^2^)/W^2^)*10 ≈ A/W^2^) [25–27], which is approximately proportional to the well-known metacarpal index, MCI = 2·T/W. It was decided to examine the RCA rather than the MCI because it is more readily comparable with other indices derived from A. In contrast, the bending breaking resistance index (BBRI) was formulated to give the fracture resistance of an idealised long bone of perfectly cylindrical shape to bending under simplifying assumptions: (W^4^−M^4^)/(W·100) ≈A·((W^2^ + M^2^)/W) [mm^3^] [28]. The BBRI is thus a special case of the section modulus known from engineering mechanics, which has also been used by other authors to describe the structural—as opposed to material—properties that co-determine bone strength [29, 30]. However an index that is to reflect bone strength relative to the bone strength requirements of an individual should as far as possible abstract from differences in body size, which neither the BMD or the BBRI does [13, 31, 32]. This was attempted with Exton-Smith’s dimensionless index, ESI = 100·T(1−T/W)/L ≈ A/(W·L) [33, 34]. Recently a further index has been presented in an attempt to improve on BMD, RCA and ESI. It is called the paediatric bone index, PBI = 10·T(1−T/W)/(W^0.33^·L^0.33^) ≈ A/(W^1.33^·L^0.33^) and is measured in μm^0.33^ [11]. It has been chosen for its low relative variability as compared with BMD, RCA and ESI in healthy Danish and Dutch children of equal bone age. This criterion is based on the assumption that, by virtue of nature’s economy, an index that reflects the ideal bone geometry in terms of strength and material efficiency should cluster more closely around the biological optimum than any other.

Our aim was to replicate the relative variability analysis mentioned above on a new database. We further wanted to extend the comparison by examining the degree to which the indices under study compensate for differences in body size. For simplicity we assumed that compensation for body size is best achieved by an index whose height dependence in children of equal age or bone age is exactly zero. This is made plausible by the fact that height is one of the two strongest determinants of bone mass [31], while, at least within its normal range of variation, it is most probably not a major determinant of bone health.

Having established by reasoning that bone strength alone is not a suitable indicator for bone health, we were interested to know how far the criterion of height independence takes us away from that of bone strength. For this purpose we looked at correlations of the BBRI with the other four indices under study. It should be stressed that this was done with descriptive intent only. We assumed that the degree of correlation with bone strength, a requirement of an ideal bone health index, is unknown and therefore cannot serve as a criterion for finding such an index.

This paper is based on data from the First Zurich Longitudinal Study (1ZLS), comprising 3,121 left-hand radiographs from 231 healthy Caucasian children ages 3 years to 19 years old. The 1ZLS has led to several publications [35–40] in which the children and the study methods have been extensively described. These children, born around 1955, grew up in a time when obesity and the trend towards sedentary activities in children did not skew normative data. The 1ZLS is to our knowledge the largest BA-related database ever used for generating normative metacarpal data. Those for T, W, M and L have been published elsewhere [40].

## Materials and methods

Informed consent was obtained from participants and parents who were present during the time of examination. The ethics committee of the University Children’s Hospital Zurich confirmed that the study was performed according to the Declaration of Helsinki and conforms to legal and ethical norms.

### Data

**Fig. 1:**
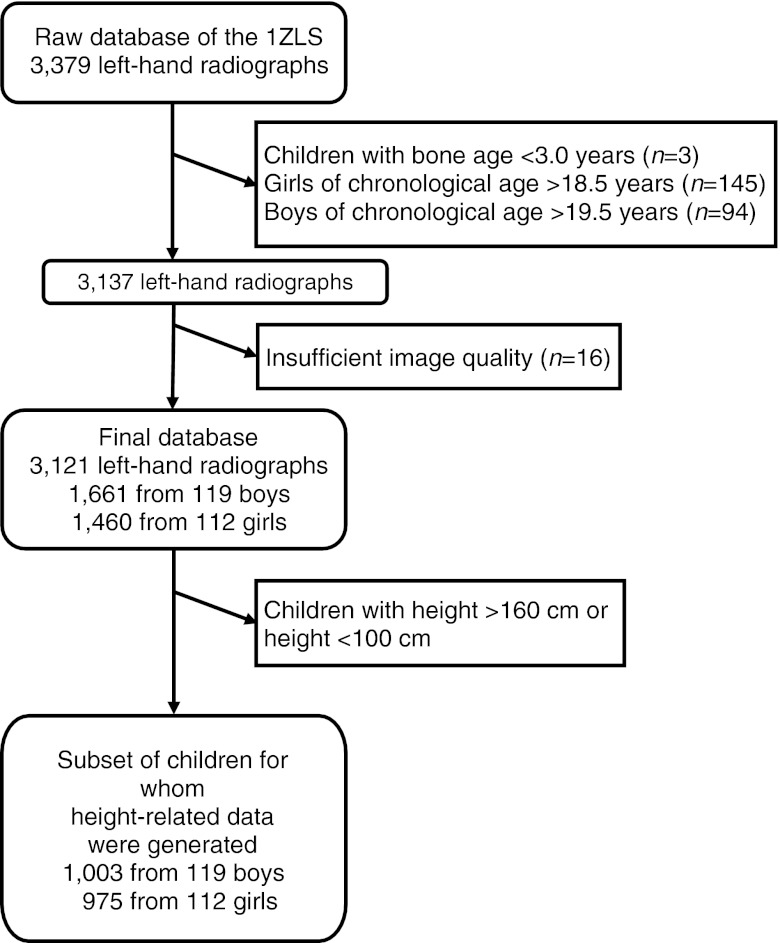
Flowchart for inclusion in the study

From a database comprising 3,379 left-hand radiographs from the 1ZLS, we selected 3,121 radiographs (1,661 from boys, 1,460 from girls) from 231 healthy Caucasian children (119 boys, 112 girls) by the following exclusion criteria: radiographs from children with bone age < 3.0 years (*n* = 3) or girls with chronological age > 18.5 years (*n* = 145) or boys with chronological age > 19.5 years (*n* = 94) or poor radiograph quality (*n* = 16). The three radiographs excluded due to bone age < 3.0 years, the only ones left in this bone age-range after removal of decayed film material, were excluded for statistical reasons. Bin sizes up to 6 years’ chronological age were also reduced due to decay of some films. The 239 radiographs from the oldest boys and girls were excluded because in this age range the 1ZLS only covered subjects who were still growing, so these would on average be skeletally delayed. The 16 radiographs excluded for quality reasons were found by screening longitudinal charts for outliers [40]. The children of the 1ZLS were born between January 1954 and February 1956 and were selected randomly within the first 2 weeks after birth [35, 36]. Girls and boys were followed until the age of 18 years and 19 years, respectively, and beyond this until their height gain was less than 0.5 cm per 2 years. Ninety-four percent of the radiographs were taken within 2 weeks of the child’s birthday and 99% within one month thereof. Data were classified by gender and chronological age, bone age or height interval. This gave 33 bins of chronological age-related and 33 bins of bone age-related data (range 3–18 years for girls and 3–19 years for boys in either case). Height-related data were only considered for children in the height-range 100–160 cm, giving 1,978 radiographs split into 24 bins (12 five-cm intervals for both genders). Due to the cut-off age, our bone age-related data are subject to a bias towards early maturers at the upper end, as explained in a study on the development of T, W, M and L in the same population [40]. This would affect bins 18–19 in boys and 17–18 in girls. Data outside the intended bone age-ranges of the software (> 17 years in boys and > 15 years in girls) should not be used for reference purposes anyway. These biased data were included in the study, since they may reveal interesting characteristics of early maturers. This was found to have no influence on the outcome of the present comparison of metacarpal indices. Figure 1 shows the exclusion criteria applied to obtain the final data sets and subsets.

### Equipment

**Fig. 2:**
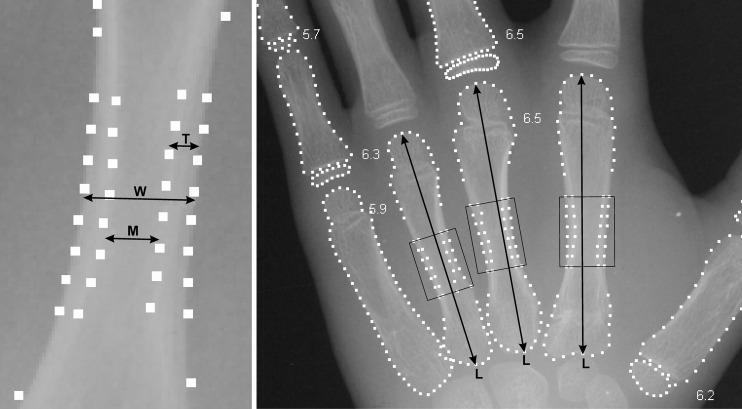
Illustration of the measurements of metacarpal cortical thickness (T), width (W), medullary diameter (M) (*left*) and length (L) (*right*). The radiograph on the right also shows the regions in which the measurements were made

The bone age and the T, W and L measurements for this study (Fig. 2) were automatically calculated by BoneXpert®, a commercially available radiograph analysis software package (Visiana, Holte, Denmark). The hardware and radiograph quality requirements of the software, its method of image processing, and the system’s compatibility with present-day clinical environments have previously been described [40]. The bone age-rating function has been validated in healthy and clinically relevant populations [38, 39, 41–44], and the method of determining T, W and L has been described elsewhere [40].

### Precision

The precision of the BA rating and radiogrammetrical functions have previously been demonstrated [37, 40]. The radiogrammetrical precision error is conventionally quoted as the precision of a single measurement, which in this case is the root mean square error (RMSE) divided by √2. Using the above-quoted precision calculation method this yielded 0.13 mm^0.33^ (2.5%) for PBI, 0.10 mm (2.8%) for BMD, 0.19 (2.9%) for ESI, 0.15 (2.3%) for RCA, and 0.16 mm^3^ (5.5%) for BBRI. These precision values were taken into account in calculating the variability of each index. The differences were small enough to have no effect on the indices’ variability ranking. The PBI discussed in this paper is implemented in BoneXpert® under the name Bone Health Index (BHI). Its measurement unit is μm^0.33^, resulting in PBI values between 3 and 7.

### Statistical analyses

Statistical analyses were performed using the JMP 7 software package of SAS (JMP, Version 9, SAS Institute Inc., Cary, NC, USA, 2010). T, W and L were computed from their mean values in metacarpals 2–4, M as W−2·T, and the mean and standard deviation of each measure was calculated for each gender by bone age-bin. The correlations of indices with bone strength were computed as Pearson correlation coefficient with the BBRI. The height-related bias of each index was determined in terms of the slope of the line of fit between the bone age-related and chronological age-related standard deviation scores (SDS) for height and the relevant index (CASDS and BASDS ratios) and expressed as SDS scores per 2 SDS of height. We used 99.8%-confidence intervals (CI) for the height-related bias of each bin to allow for 20 comparisons at significance level α = 0.05. The means and relative standard deviations of each index were calculated by gender and bone age-bin. The relative standard deviations were averaged over bone age bins 4 through 17 for girls and 4 through 18 for boys, and the resulting mean relative standard deviations were given with their ranges and standard deviations to reflect the scatter of each index. The 95%-CI was calculated for the relative standard deviations of individual randomly selected bins to gain an impression of the statistical significance of these results. The mean relative standard deviations can be used globally for any bone age-, chronological age- or height-bin to calculate Z-scores. This provides a good approximation and a considerable simplification over using a different standard deviation for each bin.

### Validation procedure

This study goes beyond the publication that first presented the PBI by considering not only the relative variability of the indices under study but also their height dependence in children of equal chronological age or bone age [11]. These two criteria are of a disparate nature and cannot be meaningfully linked by any statistical method. By considering them separately, we allowed the possibility of obtaining contradictory outcomes. Our study was thus exploratory, with collection of information being given priority over obtaining conclusive results.

## Results

**Fig. 3:**
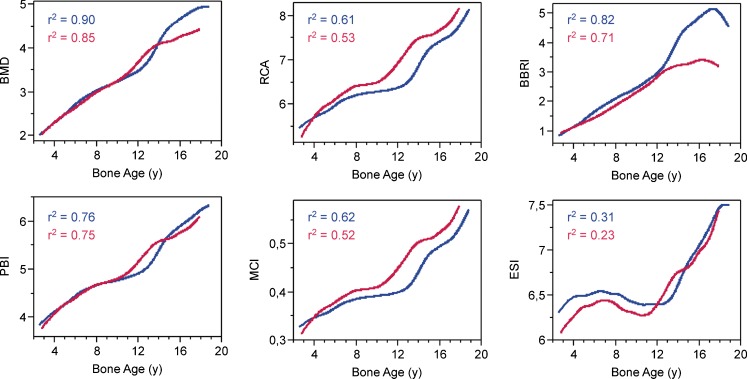
Plots of bone mineral density (BMD), relative cortical area (RCA), Exton-Smith index (ESI), paediatric bone index (PBI) and bending breaking resistance index (BBRI) versus bone age for boys (*blue*) and girls (*red*) depicted as splines (lambda =10). The coefficient of determination *r* ^2^ is given for each curve

Figure 3 shows the curves of the five indices under study: BMD, RCA, ESI, PBI and BBRI versus bone age for boys and girls. The widely used MCI has also been included to illustrate its similarity to the RCA.

**Fig. 4:**
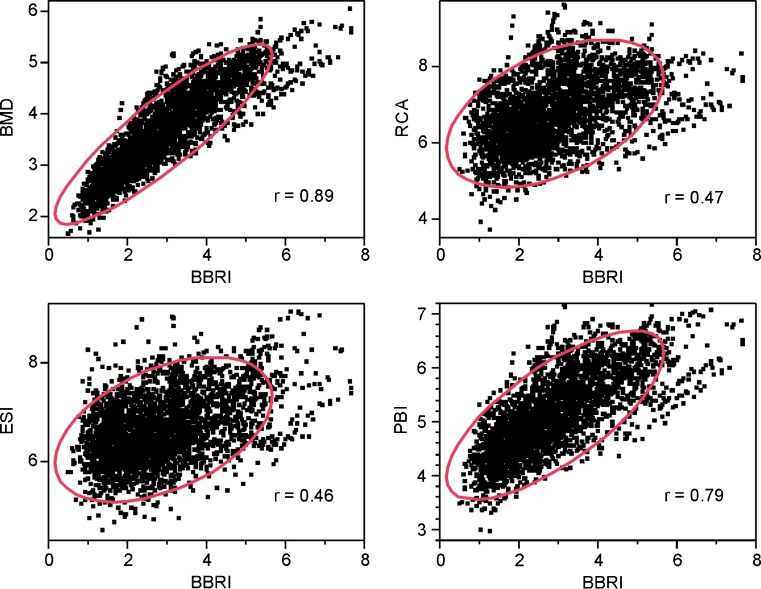
Correlations of bone mineral density (BMD), relative cortical area (RCA), Exton-Smith index (ESI), paediatric bone index (PBI) and bending breaking resistance index (BBRI) as measurements of bone strength, expressed in terms of Pearson's *r*. The ellipses contain 90% of all data points

Figure 4 shows the correlations of BMD, RCA, ESI and PBI with BBRI, which is assumed to represent bone strength. The ellipses contain 90% of all data points.

Table 1 shows the relative variability of the indices to be lowest for PBI, followed by BMD, ESI, RCA and BBRI for the genders pooled. The ranking changed when boys and girls were considered separately. There were no significant differences among the first four indices.

**Table 1:** Means and ranges of relative standard deviations (SD/mean percent) of bone mineral density (BMD), relative cortical area (RCA), Exton-Smith index (ESI), paediatric bone index (PBI) and bending breaking resistance index (BBRI), cortical thickness (T), bone width (W) and length (L), and medullary diameter (M), by gender from bone age 4 years to 17 years (girls) or 18 years (boys)

Group	Data	BMD	RCA	ESI	PBI	BBRI	T	W	L	M
Boys	Mean ± SD	7.5 ± 1.0	8.1 ± 1.2	8.3 ± 0.8	7.1 ± 0.9	17.9 ± 1.6	9.6 ± 1.0	6.6 ± 0.9	5.4 ± 0.4	12.8 ± 1.5
	Range	6.5–10.2	6.7–10.8	6.9–10.3	5.8–9.5	15.8–21.1	8.3–12.3	5.6–8.9	4.9–6.3	10.9–16.0
Girls	Mean ± SD	7.6 ± 0.6	9.5 ± 1.5	8.5 ± 0.5	7.7 ± 0.7	17.5 ± 1.4	10.7 ± 0.9	7.0 ± 0.6	5.5 ± 0.3	15.8 ± 2.2
	Range	6.6–8.5	7.5–12.5	7.8–9.5	6.7–9.3	14.1–19.9	9.2–12.1	6.3–8.0	5.0–6.2	13.3–21.1
Total	Mean ± SD	7.5 ± 0.8	8.8 ± 1.5	8.4 ± 0.7	7.4 ± 0.9	17.7 ± 1.5	10.1 ± 1.1	6.8 ± 0.8	5.5 ± 0.3	14.3 ± 2.4
	Range	6.5–10.2	6.7–12.5	6.9–10.3	5.8–9.5	14.1–21.1	8.3–12.3	5.6–8.9	4.9–6.3	10.9–21.1

Table 2 shows the height-related bias to be overall closest to zero in PBI, followed by RCA, BMD, ESI and BBRI in this order for the genders pooled, regardless of whether bone age- or chronological age-related values were considered. Both PBI and RCA showed significantly less height dependence than any of the other three indices (*p* < 0.05 for each comparison) when both genders were pooled. The outcome changed when boys and girls were considered separately.

**Table 2:** Height-related bias of bone mineral density (BMD), relative cortical area (RCA), Exton-Smith index (ESI), paediatric bone index (PBI) and bending breaking resistance index (BBRI) aggregated separately for boys and girls across bone ages 3–18 years (girls) or 3–19 years (boys) and expressed as bone age-related standard deviation scores (BASDS) per 2 height-BASDS and chronological age related standard deviation scores (CASDS) per 2 height-CASDS. (e.g. the first number, 0.53, means that BMD will on average be 0.53 SDS higher in a child with a height for bone age of 2 SDS). 99.8% confidence intervals (CI) in square brackets

Group	BMD	RCA	ESI	PBI	BBRI
BASDS					
Boys	0.53 [0.37;0.68]	0.02 [−0.14;0.18]	−0.41 [−0.56;-0.25]	0.04 [−0.12;0.20]	0.65 [0.49,0.80]
Girls	0.27 [0.11;0.44]	−0.43 [−0.60;-0.27]	−0.59 [−0.75;-0.43]	−0.30 [−0.46;-0.13]	0.89 [0.74;1.04]
Total	0.40 [0.29;0.52]	−0.20 [−0.32;-0.09]	−0.50 [−0.61;-0.38]	−0.12 [−0.24;-0.01]	0.77 [0.66-0.87]
CASDS					
Boys	0.80 [0.66;0.94]	0.11 [−0.05;0.26]	−0.27 [−0.43;-0.12]	0.24 [0.08;0.40]	0.92 [0.78;1.06]
Girls	0.49 [0.34;0.65]	−0.25 [−0.41;-0.09]	−0.43 [−0.59;-0.28]	−0.07 [−0.24;0.09]	0.99 [0.85;1.13]
Total	0.65 [0.55;0.76]	−0.07 [−0.18;0.05]	−0.35 [−0.46;-0.24]	0.09 [−0.02;0.20]	0.95 [0.85;1.05]

Table 3 gives reference data for PBI versus chronological age, bone age and 5-cm height intervals.

**Table 3:** Reference values for paediatric bone index (PBI) by bone age, chronological age, height and gender. Means and standard deviations are given in mm^0.33^. The values for bone age 18–19 years in boys and 16–18 years in girls are biased towards early maturers. Values outside the intended application range of BoneXpert® (bone-age > 17 years in boys and > 15 years in girls) should not be used for reference purposes

PBI by bone age-bin (years)				PBI by chronological age-bin (years)				PBI by height-bin (5-cm intervals)			
Bone age (years)	*N*	Mean	SD	Chronological age (years)	*N*	Mean	SD	Height (cm)	*N*	Mean	SD
Boys											
3	18	3.97	0.44	3	12	3.87	0.55	100.0–104.9	20	4.20	0.37
4	25	4.17	0.37	4	33	4.11	0.35	105.0–109.9	36	4.21	0.39
5	69	4.25	0.42	5	56	4.26	0.35	110.0–114.9	49	4.32	0.36
6	115	4.47	0.35	6	90	4.41	0.35	115.0–119.9	72	4.43	0.35
7	110	4.57	0.37	7	108	4.53	0.36	120.0–124.9	92	4.56	0.34
8	95	4.66	0.37	8	115	4.66	0.36	125.0–129.9	90	4.60	0.36
9	111	4.71	0.34	9	117	4.74	0.36	130.0–134.9	101	4.70	0.37
10	116	4.76	0.36	10	112	4.77	0.35	135.0–139.9	117	4.78	0.40
11	104	4.82	0.36	11	114	4.82	0.37	140.0–144.9	118	4.82	0.36
12	114	4.93	0.36	12	112	4.91	0.37	145.0–149.9	104	4.90	0.36
13	142	5.04	0.36	13	109	5.04	0.40	150.0–154.9	97	4.91	0.35
14	133	5.39	0.40	14	114	5.26	0.44	155.0–159.9	79	5.09	0.41
15	70	5.73	0.36	15	114	5.62	0.45				
16	81	5.88	0.42	16	114	5.86	0.43				
17	143	6.04	0.41	17	114	6.06	0.42				
18	189	6.23	0.38	18	115	6.15	0.41				
19	26	6.23	0.33	19	112	6.20	0.42				
Girls											
3	20	3.87	0.44	3	13	3.71	0.36	100.0–104.9	25	4.05	0.41
4	45	4.05	0.39	4	32	4.02	0.37	105.0–109.9	36	4.15	0.41
5	52	4.28	0.37	5	53	4.22	0.38	110.0–114.9	56	4.32	0.37
6	80	4.42	0.43	6	90	4.37	0.39	115.0–119.9	74	4.44	0.43
7	123	4.53	0.39	7	98	4.56	0.39	120.0–124.9	78	4.61	0.40
8	127	4.69	0.37	8	111	4.66	0.38	125.0–129.9	99	4.66	0.42
9	101	4.72	0.40	9	107	4.72	0.40	130.0–134.9	104	4.71	0.38
10	120	4.79	0.36	10	106	4.79	0.39	135.0–139.9	93	4.75	0.38
11	104	4.95	0.41	11	103	4.86	0.42	140.0–144.9	97	4.85	0.44
12	100	5.13	0.40	12	104	5.05	0.49	145.0–149.9	90	5.03	0.52
13	73	5.37	0.40	13	106	5.30	0.50	150.0–154.9	101	5.35	0.59
14	87	5.58	0.39	14	106	5.53	0.46	155.0–159.9	150	5.60	0.58
15	88	5.62	0.41	15	111	5.66	0.46				
16	165	5.80	0.46	16	104	5.73	0.46				
17	151	5.85	0.43	17	105	5.79	0.46				
18	24	6.01	0.44	18	111	5.84	0.46				

## Discussion

The need for an investigation of various radiogrammetrical metacarpal indices in use today is illustrated by Fig. 3. The curves of the two most widely used indices, BMD and MCI, are in particular strikingly different. An early attempt at defining a validation criterion was made by Thodberg [11], who suggested using relative variability. This was based on the assumption that when there is a choice between several parameters for describing a complex, poorly understood biological feature, the one with the lowest relative variability will indicate a feature that is important for the organism’s survival, because nature is least willing to compromise and allow wide variation. Although the differences in relative variability here are rather small, we believe that the criterion of minimal variability should in principle also apply to the field of bone physiology, especially considering the great economy with which organisms are known to make use of bone.

The BBRI showed by far the greatest relative variability of the five indices under study (Table 1). Its 95% CI per individual bin was well clear of those of any other index (not shown). Its large relative variability can also be seen intuitively from its formula, which reflects the fact that the strength of a cylindrical tube grows increasingly rapidly with increasing width. To use the concept described above, the large relative variability of the BBRI in children of equal chronological or bone age is an indication that bone health is not accurately defined by bone strength alone. The error incurred by equating the BBRI with bone strength can be neglected here because of its large deviations compared to the other indices. Its validity as a rough measure of bone strength has been studied by Gatti et al. [45], who showed DXA-BBRI of the radius to be a better predictor of femoral neck fracture than DXA-BMD measured at the same site in women. The correlations of the other four indices with the BBRI, shown in Fig. 4, thus provide a rough impression of the degree to which each reflects bone strength.

Aside from the results for the BBRI, we were unable to demonstrate significant differences between the relative variability of the indices. It was lowest for PBI and BMD, which showed virtually no difference between them, followed by the ESI and RCA. To determine whether the differences are clinically relevant, it will be necessary to review paediatric fracture studies for which hand radiographs are available. This is of particular interest at chronological age of 6–8 years due to the high incidence of low-energy fractures in this age group [46]. Table 1 also gives the relative variability of the constituent parameters (T, W, L, M) which have been discussed in greater detail previously [40].

By contrast, our results on the height-dependence of the indices were more clear-cut (Table 2). Height-dependence was overall lowest for PBI and RCA, being slightly lower for PBI in terms of bone age and, by a smaller margin, slightly lower for RCA in terms of chronological age. When the results were pooled, the difference between these two indices was not statistically significant, but both PBI and RCA showed significantly lower height-dependence than any of the other three indices, regardless of whether chronological age or bone age-related data were considered (*p* < 0.05 for each single comparison). On the other hand, by gender the differences between PBI and RCA on the one hand and the other three indices on the other were smaller, especially in girls, where bone age-related height-dependence of BMD was even slightly smaller than that of PBI and RCA. The gender differences are related to the oestrogen-induced increase in T, which is independent of height and muscle mass. These differences nearly disappeared at chronological age 6–11 years and were largest at 13–14 years.

The differences in height-dependence were large enough to be clinically relevant. In a study of 178 children with growth hormone deficiency, BMD CASDS improved from −2.2 ± 0.9 to −0.9 ± 1.1 after 4 years’ growth hormone treatment, whereas in terms of PBI, CASDS the improvement was from −1.6 ± 1.0 to −0.6 ± 1.1 (publication under preparation). This shows that height-dependence is a potentially confounding factor in metacarpal indices and needs to be taken into account. The fact that growth disorders are the main indications for radiogrammetry in children makes it all the more relevant. The large height-related bias of BMD in healthy children suggests that height-dependence should be close to zero for an ideal index of bone health. On average a normal boy with height at the tenth percentile would have a BMD BASDS of −0.43 and a BMD CASDS of −0.64 (normal girl −0.22 BASDS and −0.40 CASDS). The idea that an ideal index of bone health should have minimal height-dependence has already been discussed [47]. To eliminate the inter-gender difference in height-dependence, one would have to formulate one index for boys and one for girls. Such refinements should perhaps be deferred until the relationship between bone health and height in children has been examined in fracture studies.

Figure 4 shows that in terms of its correlation with the BBRI, the PBI has an intermediate position between the BMD and the RCA, the two indices which came closest to it by our two assessment criteria. It is tempting to speculate that this is approximately the degree of correlation with bone strength that an ideal radiogrammetrical index of bone health should have.

On comparing our data with those of the Third Zurich Longitudinal Study, with birth dates from 1973 to 1991, we found very little change in the speed of skeletal maturation but quite large deviations in metacarpal measurements. The most conspicuous change was a decrease in T. This would result in lower values for all the indices under study here. The causes underlying these very recent and very rapid changes in anthropometric measurements can hardly be identified with certainty. They may possibly be due to changes in lifestyle.

Aside from our two assessment criteria, the curves of Fig. 3 show further features that merit discussion. The decrease of BBRI versus bone age in both genders towards the end of the observation period is attributable to selection bias. The late maturers are underrepresented from bone age 17 years in boys and 15 years in girls due to our exclusion criteria. By keeping such a high cutoff, we ensured the validity of the data for the official bone age-range of BoneXpert® and of the Tanner-Whitehouse bone age system. This maturation effect is in itself interesting in that early maturers have a BBRI that is delayed for their bone age. Our outcomes remain unchanged if bone-age bins 18 and 19 in boys and 17 and 18 in girls are excluded to remove the bias towards early maturers in this range.

The reverse effect was seen in RCA, where the bias-related decrease in W versus bone age was reflected by an upward turn at the end of its curve. This highlights a weak point of the RCA. With width squared in its denominator, it produces high scores for slender bones, the opposite of what one would expect from a bone health index. Similar reservations apply to the more widely used MCI.

One feature that was particularly well highlighted by ESI was the downward slope from bone age around 7–13 years in boys and 8–11 years in girls. This reflects the dynamic changes in growth rate of T, W and L during the prepubertal dip, with T first slowing down more than W and L and then surpassing them during puberty. This is discussed in detail elsewhere [40].

Taken together our findings strengthen the case for PBI as an improved metacarpal index for children. However, it still lacks an empirical basis. An important remaining task is to test it against clinical data from children with diseases that affect bone health. This would then also allow a direct comparison of PBI and indices derived from DXA, pQCT or US. One way of refining our approach would be to consider other correction factors besides height. To determine the dependence on muscle mass, which is the other major predictor of bone mass [31], one would have to turn to other databases.

With regards to fracture risk, the convenience of being able to use hand radiographs is only a real benefit if this gives information about bones that are more prone to fracture. An extensive meta-analysis by Marshall et al. [48] suggests that increased fracture risk is most often a global skeletal condition that can be diagnosed at different skeletal sites. A decrease in bone density (or mass) by one standard deviation at any site was shown to have a similar predictive value for fractures at all sites combined [48]. Ravn et al. [49] found high correlations between BMD of the phalanges, forearm and spine measured by radioabsorptiometry, suggesting a similar conjecture with regards to the metacarpals.

Aside from the question of fracture risk prediction, the PBI can be used as a quick, inexpensive and robust method of testing for normality when taking a radiograph for assessing skeletal maturity. Especially in children, using indices based on a simple hand radiograph circumvents the serious problem of keeping patients still during pQCT and DXA examinations. For example, we have been able to show that growth hormone treatment of children with growth hormone deficiency leads to a strong and significant increase in PBI within the first year of treatment as a result of subperiosteal bone deposition [50].

## Conclusion

The paediatric bone index is less height dependent and may therefore be a better marker of pediatric bone health than area bone mineral density.
